# IL-23 signaling in Th17 cells is inhibited by HIV infection and is not restored by HAART: Implications for persistent immune activation

**DOI:** 10.1371/journal.pone.0186823

**Published:** 2017-11-01

**Authors:** Jason R. Fernandes, Tamara K. Berthoud, Ashok Kumar, Jonathan B. Angel

**Affiliations:** 1 The Ottawa Hospital Research Institute, Ottawa, Ontario, Canada; 2 Department of Biochemistry, Microbiology, and Immunology, The University of Ottawa, Ottawa, Ontario, Canada; 3 The Children’s Hospital of Eastern Ontario Research Institute, Ottawa, Ontario, Canada; 4 Division of Infectious Diseases, The Ottawa Hospital, Ottawa, Ontario, Canada; Uniformed Services University, UNITED STATES

## Abstract

**Objectives:**

HIV infection causes a profound depletion of gut derived Th17 cells, contributing to loss of mucosal barrier function and an increase in microbial translocation, thus driving systemic immune activation. Despite normalization of circulating CD4+ T cell counts with highly active antiretroviral therapy (HAART), Th17 frequency and function often remain impaired. Given the importance of interleukin (IL)-23 in the generation and stabilization of Th17 cells we hypothesized that impaired IL-23 signaling causes persistent Th17 dysfunction in HIV infection.

**Methods:**

The effects of *in vitro* HIV infection on responses to IL-23 in Th17 cells were examined. These included the production of IL-17, phosphorylated STAT3 (pSTAT3) and the transcription of retinoic acid orphan receptor C (RORC) gene. Blood derived Th17 cells from untreated and HAART-treated HIV-infected individuals were also examined for the IL-23 induced production of phosphorylated STAT3 (pSTAT3) and the expression of the IL-23 receptors.

**Results:**

*In vitro* HIV infection significantly inhibited IL-17 production and IL-23 induced pSTAT3 while expression of RORC RNA was unaffected. Th17 cells isolated from untreated and HAART-treated HIV-infected individuals showed complete loss of IL-23 induced pSTAT3 without a decrease in the expression of the IL-23 receptors.

**Conclusions:**

This study is the first to demonstrate an effect of HIV on the IL-23 signaling pathway in Th17 cells. We show that *in vitro* and *in vivo* HIV infection results in impaired IL-23 signaling which is not reversed by HAART nor is it a result of reduced receptor expression, suggesting that HIV interferes with IL-23-activated signaling pathways. These findings may explain the inability of HAART to restore Th17 frequency and function and the resulting persistent chronic immune activation observed in HIV infected individuals.

## Introduction

Among the CD4+ T cells in gut associated lymphoid tissue (GALT), the Th17 subset has been identified as a critical regulator of homeostasis and antimicrobial defense [1–3]. Found predominantly at mucosal surfaces, Th17 cells secrete a unique spectrum of cytokines that help co-ordinate adaptive and innate immune responses [4–7], and have direct effects on mucosal epithelial cells [8] that act to maintain normal mucosal homeostasis. Studies of HIV-infected individuals and SIV-infected rhesus macaques have demonstrated that the early phases of SIV and HIV infection are characterized by massive losses of Th17 cells from the GALT [9–14], facilitated by the fact that HIV preferentially infects CD4+ T cells that express the Th17 cell marker CCR6 [15]. Loss of GALT Th17 cells is associated with microbial translocation, permeability to intestinal pathogens, and damage to the mucosal epithelium [12,16–18]. Thus, Th17 deficiency is a major contributor to the systemic immune activation typical of chronic HIV infection. Despite the ability of highly-active antiretroviral therapy (HAART) to suppress viral replication and restore peripheral CD4+ T cell counts, the recovery of Th17 cells in the GALT is frequently incomplete [11,19–21].

Mouse studies have shown that terminal Th17 differentiation is dependent on chromatin remodeling of the IL-17 gene which is regulated by IL-23 [22–24], a recently described IL-12 cytokine family member. However in humans, IL-23 is believed to act by maintaining and expanding already-differentiated Th17 cells [23,25–29]. IL-23 signals through a heterodimeric receptor composed of the IL-12 receptor, beta 1 (IL-12Rβ1) chain and a unique IL-23 receptor (IL-23R) chain [30]. IL-23 signaling through its receptor requires tyrosine kinase 2 (TYK2) and Janus kinase 2 (JAK2) activity [30], and results in phosphorylation of Signal transducer and activator of transcription 3 (STAT3) which then binds to the IL-17 promoter [31–33], resulting in expression of IL-17. STAT3 phosphorylation also promotes transcription of the RAR related orphan receptor C (RORC) gene, which encodes the Th17-specific transcriptional regulators RORγt and RORα [34–36], and upregulates IL-23R and STAT3 transcription in an autocrine fashion [37,38]. Th17 cells can be programmed away from IL-17 production towards secretion of other cytokines [39–41], thus, IL-23 seems to perform a critical role in maintaining the key characteristics by which Th17 cells are identified transcriptionally and functionally.

Although HAART enables control of viral replication in the periphery, evidence suggests that viral suppression in GALT is highly variable [19]. Thus, even in well suppressed patients, ongoing viral replication in the gut may limit recovery of Th17 cells. Recently, HIV was shown to change the cytokine secretion profile of Th17 cells in the absence of overt cell death, suggesting that HIV infection may also cause Th17 dysfunction [42]. Although IL-23 has a demonstrated impact on maintaining human Th17 cell function, little is known about how HIV infection may affect the ability of IL-23 to maintain Th17 activity or key signaling pathways and transcription factors activated downstream of IL-23. We therefore sought to determine whether HIV inhibits the responsiveness of human Th17 cells to IL-23, thus contributing to ongoing Th17 deficits in HAART-treated patients.

## Materials and methods

### Study participants

All research on human blood was approved by the Ottawa Health Sciences Network Research Ethics Board. All participants provided written consent prior to participation in the study. Blood was collected from healthy volunteers, HAART-treated or untreated HIV infected individuals in heparin-containing tubes. Blood drawn from untreated individuals was collected either at a initial clinical visits at a pre-treatment time point or from individuals who had interrupted treatment. The clinical characteristics of HIV-infected patients are listed in Table 1.

**Table 1:** Clinical characteristics of HIV-infected study subjects.

	HIV-infected, untreated	HIV-infected, treated
**Median Age [range]**	43 [24–69]	45 [34–55]
		
**Gender (% male)**	75	87.5
		
**Median CD4 Count, (cells/uL) [range]**	450 [154–1,16]	471 [305–810]
		
**Median VL (log10 copies/mL), [range]**	4.4[3.1–5.6]	<1.6 [N/A]
		
**Mean** ± **SEM duration of ART (years)**	N/A	4.2 ± 1.8

### Blood Th17 cell isolation and Th17 generation from naïve CD4+ T cells

Peripheral blood mononuclear cells (PBMC) were isolated from blood by Ficoll-Paque Plus (GE Healthcare) density gradient centrifugation. Blood Th17 cells, defined as CD4+ CXCR3-CCR6+ as previously described [43,44], were isolated from PBMC by magnetic isolation using the a human Th17 cell enrichment kit according to the manufacturer’s instructions (Stemcell Technologies). Briefly, unwanted cells were extracted by negative selection using EasySep^™^ Human CD4+CXCR3- T Cell Pre-Enrichment Cocktail. Tetrameric Antibody Complexes (TAC) recognizing CD8, CD14, CD16, CD19, CD20, CD36, CD56, CD66b, CD123, TCRγ/δ, glycophorin A, CD45RAhigh, CXCR3 and dextran-coated magnetic particles, were added to PBMC and the undesired cells separated out with a magnet. Following this step which produces a highly enriched population of CD4+ T cells, CCR6+ cells were enriched by positive selection. TAC recognizing CCR6 and dextran-coated magnetic particles were added to the cells and the labeled cells then separated with a magnet. The enriched Th17 cells were then rested for 12hrs at 37 degrees to ensure the dextran-coated magnetic particles had completely dissociated from the Th17 cells. The enriched Th17 cells are known not to be activated by the enrichment process, IFN-γ and IL-17 production following the enrichment process was analyzed by StemCell and shown to be negligible without further stimulation (Magdalena Maslowski, Stem Cell, personal communication).

For some experiments, Th17 cells were generated *in vitro* from peripheral blood CD4+ T cells as previously described [45]. CD4+ T cells were isolated from PBMC using the CD4 positive selection kit (Stemcell Technologies) and activated using the T cell activation and expansion kit (Miltenyi Biotec) in RPMI-1640 supplemented with IL-1β (10 ng/mL) and IL-6 (50 ng/mL) and in the presence of neutralizing antibodies against IL-4 and IFN-γ (both at 10 μg/mL). After 5 days of priming the cells were washed and placed back into culture in RPMI-1640 supplemented with IL-2 (20 IU/mL) and IL-23 (20 ng/mL) for 7 days. All cytokines and neutralizing antibodies were purchased from R&D Systems, Inc.

## HIV infection of Th17 cells

*In vitro* HIV infection of Th17 cells was performed using a dual-tropic clinical HIV isolate grown in our laboratory (HIVCS204), or a mock preparation as a control. Cells were incubated with HIV (MOI = 0.1) for 24 hours in the absence of exogenous stimuli. Following the infection period, input virus was washed out with phosphate-buffered saline. The presence of HIV in these cultures was confirmed by PCR to detect HIV p24 DNA in infected cells. Viability of infected cultures was assessed by trypan blue exclusion and found to be >85% at time of analysis.

### IL-17 intracellular cytokine staining

Th17 cells were stimulated with PMA (5 ng/mL) (Sigma-Aldrich) and ionomycin (50 ng/mL) (Sigma-Aldrich) in RPMI-1640 for 6 hours, in the presence of the protein transport inhibitor brefeldin A (Golgiplug) as per the manufacturer’s instructions (BD Biosciences). Following stimulation the cells were washed with 1% BSA-PBS and fixed and permeabilized using the Invitrogen Fix & Perm kit, according to the manufacturer’s instructions. Permeabilized cells were stained for 30 minutes at room temperature with anti-IL-17-Alexa Fluor 647 (BD Biosciences). Excess antibody was washed out and the cells were resuspended for analysis. A minimum of 30,000 events per condition were acquired on an FC500 flow cytometer (Beckman Coulter). Negative fluorescence cut-offs were defined using fluorescence minus-one (FMO) controls.

### Measurement of cytokine secretion

HIV-infected or uninfected Th17 cells were activated with beads coated with monoclonal antibodies against CD3 and CD28 in the presence of IL-23 (20 ng/mL). Supernatants, collected following 3 days of activation and secretion of IL-17 by Th17 cells, were measured using the Ready-Set-Go IL-17 ELISA (R&D Systems). Developed ELISA plates were read using a SpectraMax 190 (Molecular Devices). Cytokine concentrations were determined using the regression line from a standard curve performed in triplicate with each assay. All samples were assayed in triplicate and IL-17 concentrations were expressed as ng/mL, mean ± SEM.

### STAT3 phosphorylation analysis

For the detection of phosphorylated STAT3 (pSTAT3), enriched Th17 cells were cultured in RPMI-1640 alone or in the presence of either IL-23 or IL-6 (both at 50 ng/mL; R&D Systems) for 15 minutes at 37°C. The incubation time of 15 minutes was determined to be the optimal time for pSTAT3 expression. Incubation times longer than 15mins were not shown to further enhance the pSTAT3 detection in either HIV+ve or HIV-ve Th17 cultures. Following stimulation the cells were immediately fixed by the addition of paraformaldehyde at 37°C for 10 minutes. The cells were then washed and permeabilized on ice for 30 minutes using BD Phosflow buffer III (BD Biosciences). Following permeabilization, the cells were washed and stained with anti-pSTAT3-AlexaFluor 488 (BD Biosciences) for 30 minutes at room temperature, then washed, and resuspended for analysis. A minimum of 30,000 events in the lymphocyte scatter gate was collected for each sample on an FC500 flow cytometer (Beckman Coulter). Negative fluorescence cutoffs were defined using unstimulated controls. Data presented represent the proportion of cells that stain positive for pSTAT3.

### qRT-PCR

RNA was isolated from cells using the RNeasy mini kit (Qiagen) and reverse transcribed into cDNA using the Invitrogen Superscript III First Strand Synthesis kit (Invitrogen). qRT-PCR reactions were set up using commercially available primers for the human RORC gene (Qiagen). *18S* rRNA was amplified using the following specific primers: 5’-CTGCCATTAAGGGTGTGG-3’ [forward] and 5’-TCCATCCTTTACATCCTTCTG-3’ [reverse]. Reactions were prepared with iQ SYBR green supermix (Bio-Rad). Amplification was assessed by measuring the fluorescent signal of SYBR green using an iCycler Real-time PCR thermocycler (Bio-Rad). mRNA levels were calculated using the ΔΔCt method, normalized to *S18* rRNA. Results are expressed as fold change relative to unstimulated, uninfected cells.

### IL-23R staining

For the flow cytometric detection of IL-23R Th17 cells, enriched with the StemCell Th17 enrichment kit as previously described, were fixed in paraformaldehyde at room temperature for 20 minutes. The cells were washed and permeabilized in 0.05% saponin in PBS. IL-23R was stained using a rabbit polyclonal antibody (EMD Millipore), which stains for the cytoplasmic domain of the IL-23R, or a rabbit IgG isotype control (Jackson Immunoresearch) for one hour at room temperature. Excess antibody was washed out, and the bound primary antibody was stained using a donkey-anti-rabbit-AlexaFluor 488 conjugate for one hour at room temperature (Life Technologies). Excess antibody was washed out, and the cells were resuspended in PBS/BSA buffer for analysis. A minimum of 10,000 events in the lymphocyte gate were collected using an FC500 flow cytometer (Beckman Coulter). Negative cut-offs were defined using isotype controls.

### Western blots

For detection of the IL-23 receptor protein IL-12Rβ1, blood Th17 cells were lysed in RIPA buffer in the presence of protease inhibitors for 30 minutes on ice, and centrifuged at 20,000 x g for 30 minutes at 4°C. Lysates were separated on a 12% polyacrylamide gel and transferred to PVDF membranes. IL-12Rβ1 protein was labeled using a monoclonal antibody (Santa Cruz Biotechnology) and detected with horseradish peroxidase-conjugated goat anti-rabbit IgG (R&D Systems). β-actin was similarly detected for use as a loading control. Images were acquired using an AlphaImager imaging system and AlphaView software (ProteinSimple).

### Statistical analysis

Differences between *in vitro* infected/uninfected groups were tested using paired two-tailed Student’s t tests. Comparisons of healthy control and HIV patient data were tested using one-way ANOVA and Tukey’s multiple comparison tests. Data was analyzed using Graphpad Prism software v 6.0, with a P level < 0.05 considered to be statistically significant.

## Results

### In vitro HIV infection reduces IL-17 secretion and expression by Th17 cells

**Fig 1:**
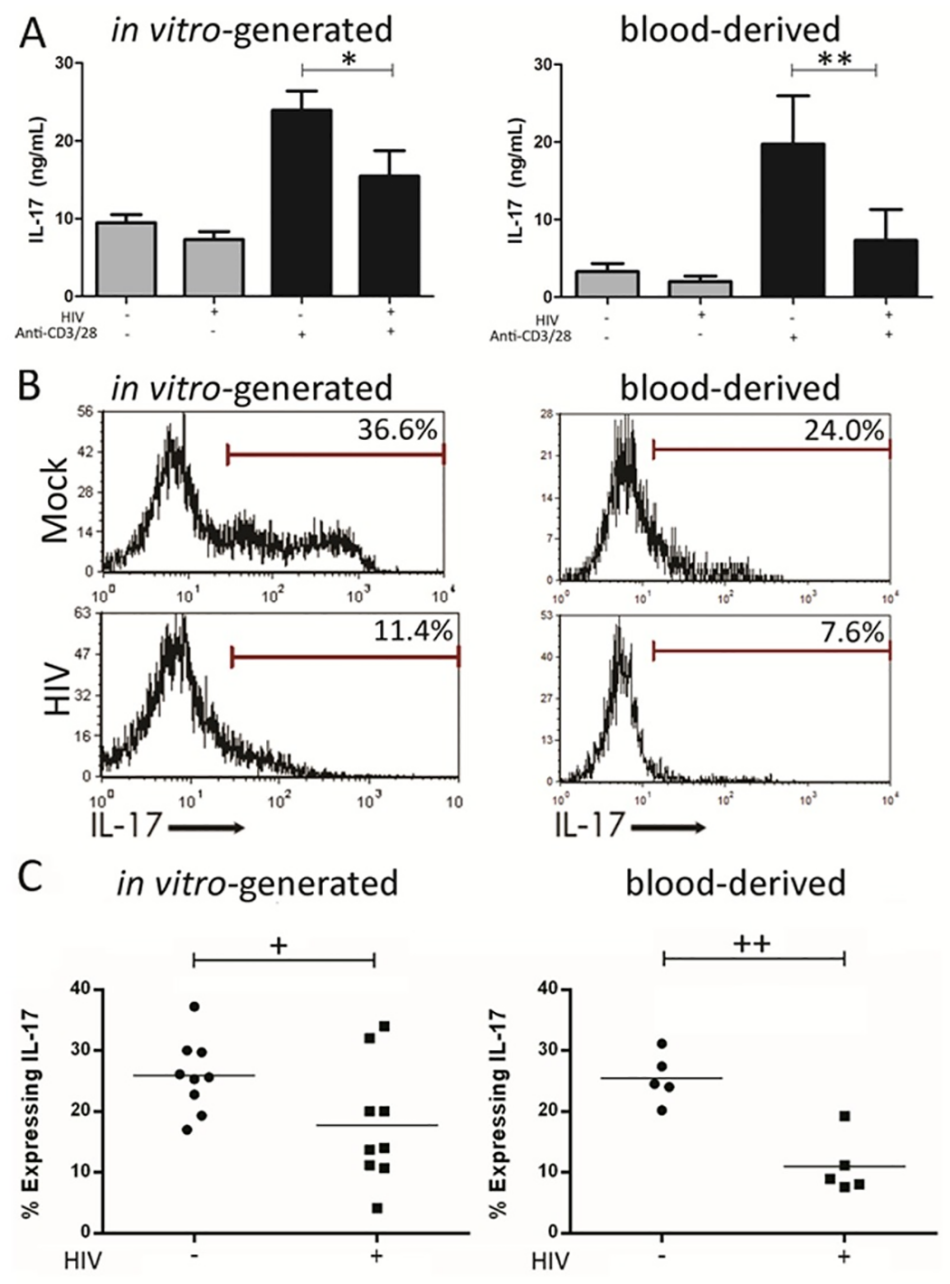
HIV reduces IL-17 secretion and intracellular expression in blood-derived and *in vitro*-generated Th17 cells. Blood-derived and *in vitro* generated Th17 cells were infected with HIV for 24 hours. **(A)** Th17 cells were cultured for 3 days with anti-CD3 and anti-CD28 mAbs. Supernatants were harvested and assayed for secreted IL-17 by ELISA; * p < 0.001, n = 9; ** p = 0.045, n = 9. Data shown are mean ± SEM. **(B)** Representative histograms showing intracellular expression of IL-17 in Th17 cells stimulated for 6 hours with PMA and onomycin in the presence of brefeldin A. (**C)** Summary of the proportion of blood-derived and *in vitro* generated Th17 cells expressing IL-17 following PMA and ionomycin stimulation is shown with mean frequency indicated; + p = 0.026, n = 9, ++ p = 0.012, n = 5.

We examined the effects of *in vitro* HIV infection on the production of the canonical Th17 cytokine IL-17 by Th17 cells. Spontaneous IL-17 secretion by blood-derived Th17 enriched cells and *in vitro*-generated Th17 cells was minimal and was not significantly altered by HIV infection (Fig 1A). Infection with HIV significantly reduced anti-CD3/anti-CD28-induced secretion of IL-17 by blood-derived (p < 0.001) and *in vitro*-generated (p = 0.046) Th17 cells (Fig 1A). HIV-infected Th17 cells were stimulated with PMA/ionomycin in the presence of brefeldin A to determine the effect of HIV infection on the frequency of IL-17+ cells. Infection with HIV significantly impaired PMA/ionomycin induced expression of IL-17 by *in vitro*-generated Th17 cells (p = 0.013, Fig 1B/1C). Similar results were observed for blood-derived Th17 cells (p = 0.026, Fig 1B/1C). These results indicate that even short-term exposure to HIV impairs IL-17 expression by Th17 cells.

### HIV infection inhibits IL-23 induced phosphorylation of STAT3 in Th17 cells

IL-23 has been identified as a key cytokine for the generation and maintenance of human Th17 cells, and it has been hypothesized that lack of IL-23 signals leads to loss of Th17 function, and in particular the propensity to secrete IL-17[23,25–29]. Phosphorylated STAT3 (pSTAT3) is the primary transcription factor associated with IL-23 signaling in Th17 cells [31–33]. Phosphorylated STAT3 can also be induced through the binding of IL-6 to its receptor [46]. Therefore, to determine whether HIV infection influences IL-23 signaling in Th17 cells, HIV-infected cells were stimulated with IL-23 or IL-6 and pSTAT3 was assessed by flow cytometry.

**Fig 2:**
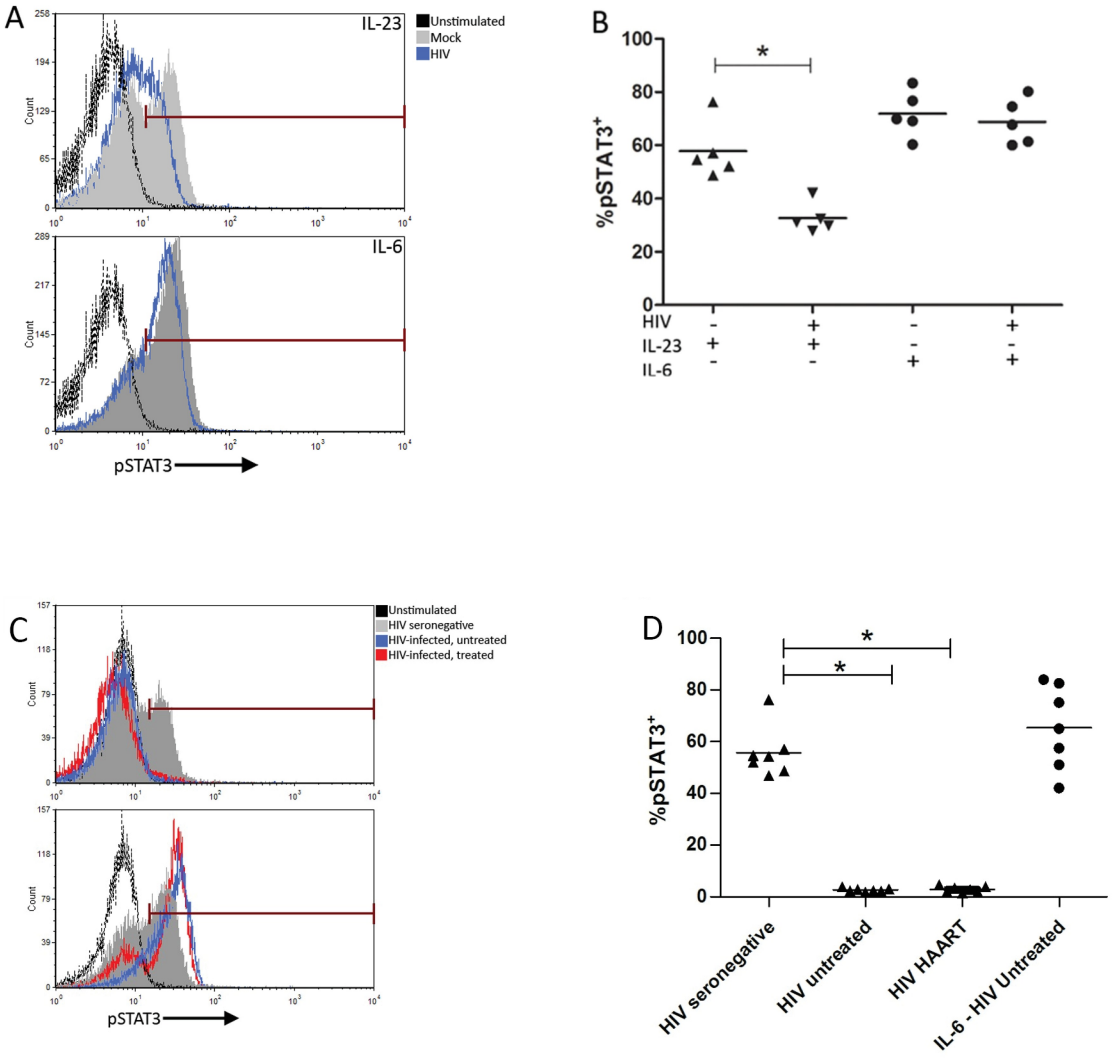
Phosphorylation of STAT3 within Th17 cells in response to IL-23 is inhibited by *in vitro* and *in vivo* HIV infection and is not restored by HAART. Blood-derived Th17 cells from healthy donors were infected with HIV for 24 hours. Input virus was washed out, and cells were stimulated with IL-23 or IL-6 for 15 minutes. **(A)** Representative histograms showing STAT3 phosphorylation in response to IL-23 or IL-6 in HIV-infected and uninfected Th17 cells. **(B)** Summary of frequency of Th17 cells responding to IL-23 or IL-6 by phosphorylation of STAT3 (%pSTAT3+). * p = 0.001, n = 5. **(C)** Representative histograms demonstrating STAT3 phosphorylation following IL-23 (top) and IL-6 (bottom) stimulation in Th17 cells isolated from patients and healthy controls. **(D)** Summary of IL-23 induced pSTAT3 responses in circulating Th17 cells from HIV seronegative, HIV-infected untreated and HIV-infected HAART donors (* p < 0.001, n = 7) and IL-6-induced pSTAT3 responses in Th17 cells isolated from HIV infected donors.

Unstimulated cells expressed a negligible amount of pSTAT3 (1.8 ± 0.1% of cells stained positive for pSTAT3). Stimulation of uninfected Th17 cells with IL-23 induced a robust pSTAT3 response (57.8 ± 4.8%), was significantly reduced by *in vitro* HIV infection (32.7 ± 2.5%, p = 0.001, Fig 2A and 2B). In contrast, Th17 responsiveness to IL-6, which also induces pSTAT3, was comparable between uninfected (71.9 ± 3.9%) and HIV-infected Th17 cells (68.8 ± 3.9%, Fig 2A).

### Th17 cells from HIV-infected individuals do not express phosphorylated STAT3 in response to IL-23

Having demonstrated that HIV interferes with IL-23 signaling in Th17 cells infected with HIV *in vitro*, we next examined whether circulating Th17 cells from HIV infected patients displayed similar IL-23 signaling defects. Circulating Th17 cells were enriched with the STEM cell Th17 enrichment kit and stimulated for 15 minutes with IL-23 or IL-6 and pSTAT3 was assessed by flow cytometry. IL-23 induced pSTAT3 was completely absent in circulating Th17 cells from untreated HIV-infected patients (2.1 ± 0.3% vs. 57.8 ± 4.8% in uninfected controls, p < 0.001 Fig 2C and 2D), suggesting that *in vivo* HIV infection interferes with the initial events in IL-23 signaling. Surprisingly, IL-23 induced pSTAT3 responses within circulating Th17 cells from HAART-treated patients was similarly diminished (3.7 ± 0.6%, p < 0.001; Fig 2C and 2D). Responses to IL-6 were not significantly different among untreated patients, treated patients and uninfected controls (65.3 ± 6.1%, 68.1 ± 9.8% and 73.2 ± 2.8%, respectively). These results are consistent with the *in vitro* results and demonstrate that HIV infection results in selective interference with IL-23 signaling in human Th17 cells while the cellular phosphorylation machinery related to STAT3 remains intact.

### In vitro HIV infection has no effect on RORC expression

**Fig 3:**
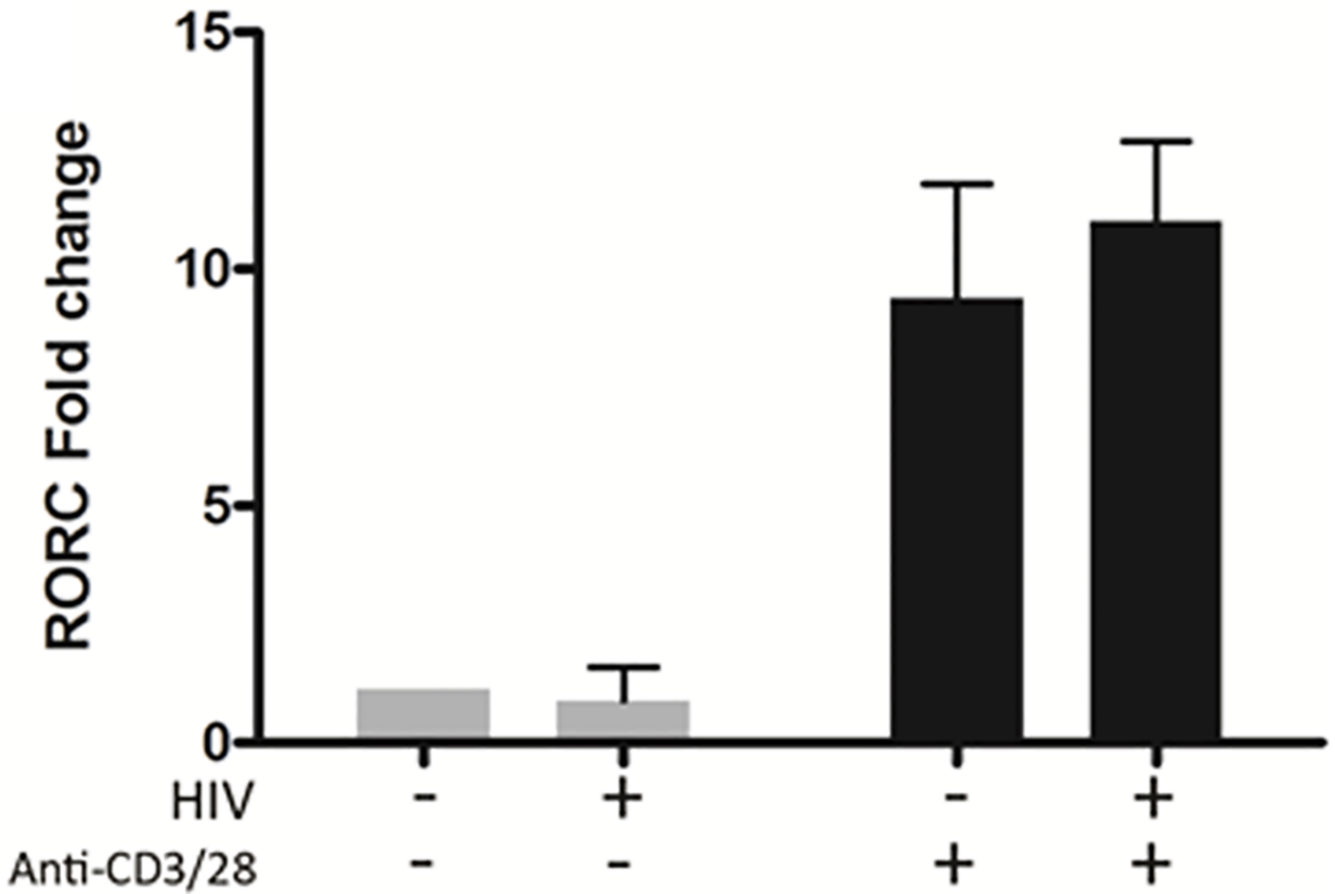
*In vitro* HIV infection does not affect RORC, RNA levels in blood-derived Th17 cells. Blood-derived Th17 cells were infected with HIV for 24 hours. Input virus was washed out and cells were stimulated with anti-CD3 and anti-CD28 mAbs for 3 days. mRNA was isolated and quantified by real-time qPCR for RORC genes. Relative mRNA levels were calculated using the ΔΔCt method, normalized to 18S rRNA. Results are expressed as fold change relative to unstimulated, uninfected cells. n = 9.

Central Th17 transcription factors RORα and RORγt are encoded by the RORC gene, the transcription of which is triggered by anti-CD3/CD28 stimulation [47]. To identify whether HIV inhibits expression of RORC, blood-derived Th17 cells were infected with HIV and stimulated for 3 days with beads coated with monoclonal antibodies against CD3 and CD28. Relative expression of RORC mRNA was determined by qRT-PCR. The transcription of RORC was not affected by infection with HIV (Fig 3).

### IL-23 receptor expression is not downregulated in HIV infection

**Fig 4:**
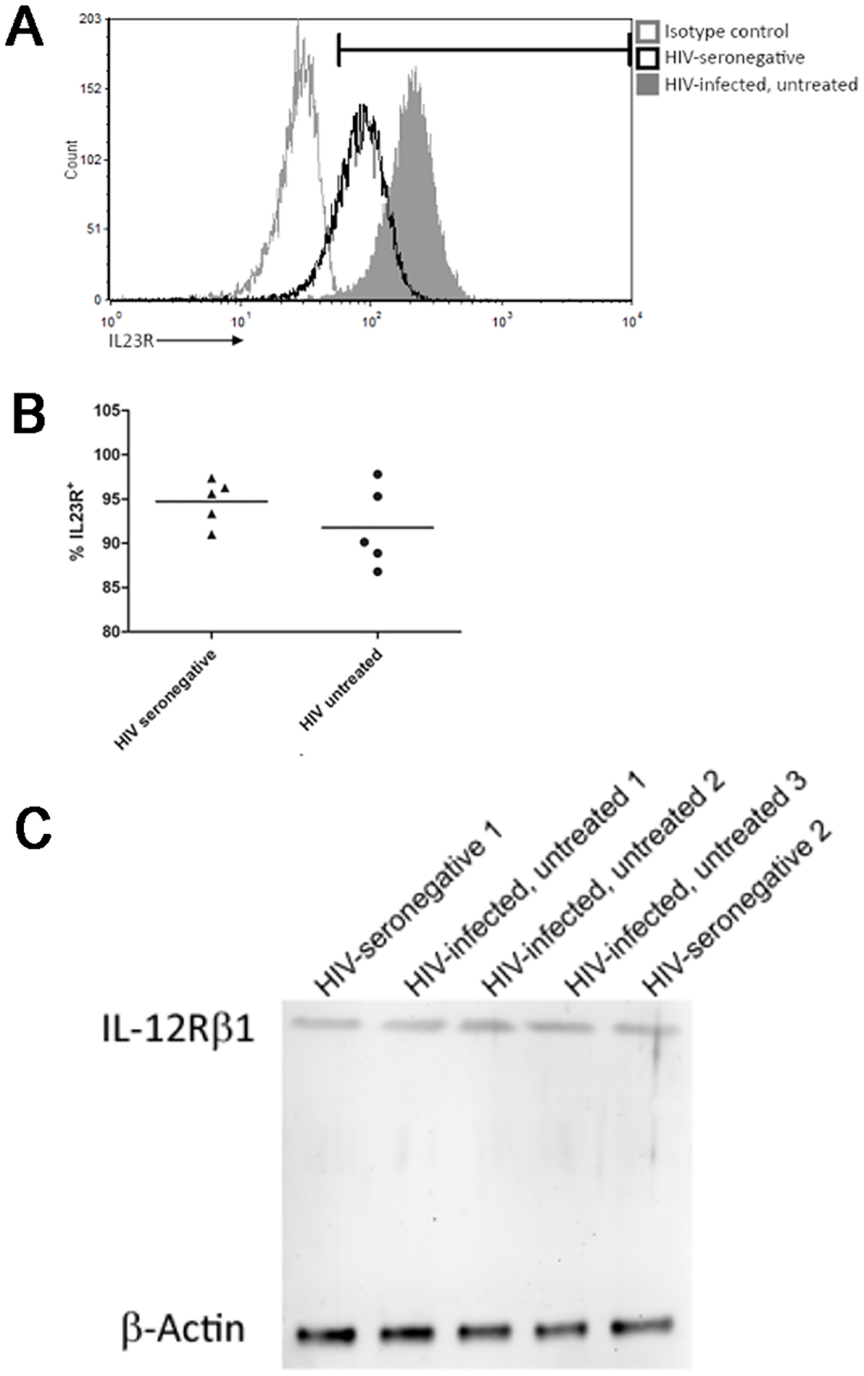
HIV infection does not downregulate IL-23R expression. Circulating Th17 cells were isolated from HIV-seronegative or untreated HIV-infected individuals and expression of IL-23R was assessed by flow cytometry. **(A)** Representative histograms demonstrating IL-23R expression on circulating Th17 cells isolated from HIV-seronegative controls, HIV-infected untreated, HIV-infected HAART treated individuals and the matched IL-23R isotype control. **(B)** Summary of % cells expressing IL-23R on circulating Th17 cells from HIV seronegative and untreated HIV-infected patients. **(C)** Western blot demonstrating expression of IL-12Rβ1 on blood Th17 cell lysates from HIV-seronegative and HIV infected, untreated donors. Figure is representative of 3 out of 6 donors tested.

The IL-23 receptor complex is a heterodimer composed of the IL-12 receptor beta 1 chain (IL-12Rβ1) and a unique polypeptide chain called IL-23R [30]. HIV infection has previously been shown to alter cytokine responsiveness through multiple mechanisms [48–51], and so it is possible that the observed hyporesponsiveness to IL-23 was due to downregulation of one or both of the receptor chains on circulating Th17 cells. In HIV-seronegative donors, nearly all circulating Th17 cells were found to express IL-23R (95.1 ± 1.4%), comparable to values observed in untreated HIV infected individuals (92.2 ± 2.6%) (Fig 4B). No decrease in the geometric mean fluorescence intensity (MFI) of the IL-23R expression on Th17 cells was found in the HIV infected untreated donors compared to the HIV seronegative donors. Expression of the IL-12Rβ1 chain was similarly unaffected in HIV seropositive individuals (Fig 4C). Taken together, these observations confirm that HIV-induced defects in IL-23 signaling are not due to lack of IL-23R or IL-12Rβ1 expression.

## Discussion

While Th17 cell expression and function are impaired in HIV infection, the mechanisms by which this occurs are yet to be identified. Here we describe for the first time the effect of HIV on the IL-23 signaling pathway in Th17 cells. The principle findings of this study were as follows: (1) expression of the canonical Th17 cytokine IL-17 is significantly inhibited by *in vitro* HIV infection; (2) both *in vivo* and *in vitro* HIV infection significantly reduce IL-23-induced pSTAT3 expression which is not reversed in the setting of effective HAART; and (3) the observed reduction in IL-17 is not due to the effect of HIV infection on RORC mRNA expression in Th17 cells nor its effect on the IL-23 receptor expression. The apparent irreversibility of the IL-23 signaling defects with effective HAART underscores the challenges in restoring mucosal homeostasis in HIV infected individuals and may explain in part why immune activation persists despite suppression of viral replication.

IL-17 production in response to TCR (anti-CD3/CD28) stimulation was shown to be significantly impaired by *in vitro* HIV infection. This is consistent with previous reports indicating that HIV proteins can cause reductions in CD3/CD28 activation of CD4+ T cells. In particular, the HIV accessory protein Nef interferes with the strength of CD3-mediated signaling [52–54], potentially providing an explanation for the observed reduction in IL-17 secretion in response to TCR stimulation. However, this does explain the reduction in IL-17 expression induced by PMA/ionomycin, which bypasses the need for TCR activation.

HIV infection significantly reduced IL-23 induced pSTAT3 responses in both Th17 cells isolated from HIV infected individuals and HIV infected Th17 cultures. When stimulated with IL-6, the pSTAT3 responses in Th17 cells remained intact. These robust pSTAT3 responses to IL-6 indicate that STAT3 protein is present and functional within the *in vitro*-infected Th17 cells, as well as the Th17 cells isolated from HIV patients. A greater reduction in the pSTAT3 responses to IL-23 was detected in the Th17 cells isolated from HIV infected patients compared to the Th17 cells cultured with HIV (Fig 2B and 2D). Host and environmental factors such as the cytokine milieu may have contributed to the differences seen.

The IL-23 receptor (IL-23R and IL-12Rβ1) also remained unaffected by HIV infection indicating that receptor downregulation is not the cause of impaired IL-23 responses. HIV may therefore be interfering with intracellular signaling events downstream of the IL-23 receptor, but upstream of STAT3 phosphorylation.

On binding to the IL-23 receptor complex, IL-23 induces the phosphorylation of JAK2 and TYK2 with which the IL-23 receptor is constitutively associated [30], leading to the phosphorylation of STAT3 [55]. It is possible that HIV may interfere with the phosphorylation of JAK-2 and TYK-2 proteins and explain the reduction of IL-23 induced pSTAT3 we observed. However while IL-23 has been shown to induce the phosphorylation of JAK-2 and TYK-2 in human CD4 T-cell clones [30], IL-23 induced phosphorylation of JAK-2 and TYK-2 has not been documented in human primary Th17 cells. In fact, in our model, IL-23 was unable to induce phosphorylation of JAK-2 and TYK-2.

HIV may also induce negative regulators of the IL-23 signaling pathway. Recent studies in rhesus macaques by Bixler SL et al. showed that during acute SIV infection, IL-17 mRNA expression in CD4+ T cells was significantly reduced. This reduction was correlated with the increased expression of the negative regulators: protein inhibitor of activated STAT3 (PIAS3), Src Homology Phosphatase 2 (SHP2) and suppressor of cytokine signaling 3 (SOCS3)[56]. HIV infection has also been shown to induce SOCS1 and SOCS3 mRNA in CD4+ T cells in HIV infection [49], and the HIV trans-activator protein (tat) impairs IFN-γ expression by inhibiting STAT1 activation via a SOCS2-dependent pathway [57]. Thus, induction of negative regulators may contribute to an impaired potential to produce IL-17, though IL-23 is not known to induce such negative regulation in Th17 cells.

The RORC gene products, RORγt and RORα, promote Th17 differentiation [58] and IL-17 production [59]. Although RORC mRNA levels are not altered by HIV infection, it is still possible that HIV induces protein-protein interactions that limit the activity of RORC gene products. In mice, the protein FoxP3 has been shown to inactivate RORγt [60] and a similar interaction has been described for RORα [61]. In humans, HIV infection has been shown to induce higher levels of FoxP3 expression in CD4+ T cells. HIV infection has also been shown to increase STAT1 levels in human T-cells [62], and to induce STAT1 activation in monocyte-derived macrophages and human brain microvascular endothelial cells (HBMECs) [63,64]. STAT1 induction then leads to the inhibition of RORγt via the transcription of T-bet. Thus HIV infection may induce the expression of host proteins that antagonize transcription factors involved in the control of Th17 differentiation and function.

In the context of HIV infection, persistent immune activation has been implicated in much of the morbidity that remains despite the effectiveness of HAART. Studies have demonstrated that when Th17 reconstitution has been observed, a reduction in microbial translocation and systemic immune activation markers is also observed [11,65–67], and that preservation of intestinal Th17 cells in SIV-infected rhesus macaques is associated with reduced microbial translocation and systemic immune activation [68]. Consistent with the hypothesis that HIV alters Th17 function and generation, we have shown that Th17 cells lose the ability to express IL-17 following infection with HIV. Both *in vitro* and *in vivo* HIV infection significantly inhibit the ability of Th17 cells to respond to IL-23, a key cytokine implicated in the generation of Th17 cells and the maintenance of their function. Importantly, this lack of responsiveness to IL-23 does not appear to be reversed by HAART. This irreversible defect in IL-23 signaling in Th17 cells may be a driving factor for microbial translocation and persistent immune activation, and thus should be a focus for therapeutic targets to reduce immune activation-associated morbidity in HIV infection.
